# Exploring the effects and mechanisms of organophosphorus pesticide exposure and hearing loss

**DOI:** 10.3389/fpubh.2022.1001760

**Published:** 2022-11-11

**Authors:** Mingshan Zou, Mao Huang, Jianyun Zhang, Rong Chen

**Affiliations:** Department of Preventive Medicine, School of Public Health, Hangzhou Normal University, Hangzhou, Zhejiang, China

**Keywords:** organophosphorus pesticides, sensorineural hearing loss, oxidative stress, DNA damage, inflammatory response

## Abstract

Many environmental factors, such as noise, chemicals, and heavy metals, are mostly produced by human activities and easily induce acquired hearing loss. Organophosphorus pesticides (OPs) constitute a large variety of chemicals and have high usage with potentiate damage to human health. Moreover, their metabolites also show a serious potential contamination of soil, water, and air, leading to a serious impact on people's health. Hearing loss affects 430 million people (5.5% of the global population), bringing a heavy burden to individual patients and their families and society. However, the potential risk of hearing damage by OPs has not been taken seriously. In this study, we summarized the effects of OPs on hearing loss from epidemiological population studies and animal experiments. Furthermore, the possible mechanisms of OP-induced hearing loss are elucidated from oxidative stress, DNA damage, and inflammatory response. Overall, this review provides an overview of OP exposure alone or with noise that leads to hearing loss in human and experimental animals.

## Background

According to the latest World Health Organization (WHO) report, hearing loss currently affects more than 1.5 billion people in the world, accounting for 20% of the global population. About 1.16 billion people present mild hearing loss, while 430 million people (5.5% of the global population) have moderate to severe or greater hearing loss (1). It is estimated that over 900 million people will suffer from disabling hearing loss by 2050. Hearing disability accounts for 24.16% of the total number of disability in China, which places a heavy burden on society and families. The prevalence of hearing loss with more than 15 decibels (dB) in one or both ears has increased dramatically among children (2). About 34 million children suffer from hearing loss, which has been increasing since 1990 (3). In China, approximately 20,000–30,000 hearing-impaired children are born every year, while 120,000 children developed severe to profound hearing loss before the age of 7 years (4). A report from Australia shows that nearly 10% of children aged 11–12 years have a bilateral hearing loss of more than 16 dB at the main speech frequencies, with a majority having hearing impairment of a slight degree (16–25 dB HL). Unilateral losses are quite prevalent, while hearing loss at a lower frequency is more common than that at a higher frequency (3). Hearing loss affects learning, work, and quality of life in children or adults and is related to reduced social communication, emotional instability, and cognitive dysfunction (5). A variety of factors, such as genetics, exposure to environmental risk factors, complications at birth, certain infectious diseases, chronic ear infections, noise exposure, ototoxic drug, and aging, contribute to hearing loss, some of which simultaneously have a superimposed effect and lead to an increased likelihood of hearing loss.

Pesticides are a broad group of chemical or biological agents that deter, incapacitate, kill, or control undesired pests, including insecticides, fungicides, rodenticides, pediculicides, and biocides (6). Among them, organophosphorus pesticides (OPs) are widely used in agriculture, horticulture, and many other fields, owing to their advantages of better insecticidal efficacy and higher safety profiles. According to statistics, the consumption of OPs was the highest, accounting for approximately 33% [about 748 million kilograms (kg)] of worldwide pesticide usage (7, 8). China is the world's top producer of pesticides, of which approximately 70% are attributed to OPs (9). Over 3.0 billion kgs of OPs are annually applied as pesticide in agriculture in China (10). The most commonly used OPs include parathion, malathion, chlorpyrifos (CPF), and diazinon (11). The extensive use of OPs has caused a vast degree of pollution to the environment. Even the WHO classifies OPs as extremely dangerous chemical compounds (12). Hence, the restricted use of moderately toxic OPs is followed in some areas. For example, parathion with high toxicity and high residue has been banned for use on crops in the European Union (EU), Australia, and China. CPF has been banned for use on crops used in poultry farming and other crops in the USA as well as on vegetables during vegetable cultivation in China since 2014. CPF had reached the highest usage of approximately 10 million kg per year from 1982 to 2016, even though the lowest use of CPF came up to as much as 7.4 million kg in 2004, according to the study by Derbalah A et al. on the risk assessment of OPs in Japan. OP residues were detected at unacceptable levels in several rivers in Japan (13). In natural wetlands in the Mexico City metropolitan area, the OPs exhibit a high bioaccumulation pattern in the accumulating plant-water hyacinth (14); OPs and their metabolites have been widely detected in crops, water sources, and soils (15). For example, the metabolite 3,5,6-trichloro-2-pyridinol (TCP) of CPF has a higher water solubility and a wider range of contamination in soil and water (16–18). Those details highlighting the harmful effects caused by OPs mean that the OPs are transported to and get accumulated in the environment, bringing about continuous pollution to the environment throughout the world.

Meanwhile, OP poisoning is a major global clinical problem in developing countries with the highest incidence and a high lethality rate. Pesticide residues in food and air constitute the main chronic exposure routes for the general population (19, 20). A study of children's diets and their exposure to OPs in Washigton showed that the metabolites of CPF and malathion significantly decreased to non-detectable levels in urine tests when organic foods were consumed, instead of conventional foods (21). In addition, OP exposure can also arise from dust residues, residential pest control, or aerial spraying to kill mosquitoes, etc. (22). Prenatal exposure to OPs has been well established to present a negative impact on the neurological development of infants, as well as neurotoxicity in adults and animals, by irreversibly binding to acetylcholinesterase (AChE), thus affecting the neurological function (23–25). Moreover, epidemiological studies showed chronic exposure to low doses of OPs and their metabolites, which is associated with adverse health effects for humans, such as high genotoxicity, liver dysfunction, embryotoxicity, neurotoxicity, interference with vitamin D3 metabolism, obesity, diabetes, etc. (26).

Currently, a neuroprotective drug is used for the common treatment of acute OP poisoning clinically, but few measures on mitigating the potential risk of hearing loss caused by OPs have been undertaken (27). This is likely due to fewer concerns about the effects of OP exposure and hearing loss. Hearing loss is a permanent sensory disorder. It is important to prevent hearing loss before it occurs, as recovery is nearly impossible. Considering the widespread usage of OPs and their potential health risks, this study provides the latest evidence to clarify the possible effects of OPs on hearing loss through epidemiological population studies and experimental animal models and further explores their possible toxic mechanisms.

## Population studies

Population studies are conducive to describing the disease burden and identifying its potential risk factors, further offering an evidence base for mechanistic studies. The effects of OP exposure on the auditory system described in population studies are summarized in Table 1. For an objective assessment of the hearing function, otoacoustic emissions (OAE) testing was performed to determine the micromechanics status of hair cells (HCs), and an auditory brainstem response (ABR) testing was conducted to evaluate the integrity of the cochlea, the auditory nerve, and central transmission (28). The Agricultural Health Study initiated in America evaluated the relationship between hearing loss and pesticide exposure among licensed pesticide applicators in Iowa and North Carolina from 1993 to 1997. The results showed that hearing loss was positively associated with licensed pesticide applicators exposed to OPs (odds ratio (OR) = 1.17; 95% confidence interval (CI) = 1.03–1.31) (29). Tomiazzi et al. showed that some farmers in Brazil faced symptoms of hearing difficulties or persistent bilateral tinnitus; and the groups exposed to insecticides (including methyl parathion, CPF, and diethyl succinate) included a greater number of individuals with numerical changes in high-frequency hearing compared with controls (*p* < 0.05) (30, 31). A cross-sectional study was conducted by Choochouy et al. in three agricultural regions in Thailand. Controlling confounders of age, smoking, and alcohol consumption, the synergistic effects of cumulative exposure time to pesticides (OPs, carbamates or cypermethrin, herbicides, and fungicides) and cumulative noise exposure affected high-frequency hearing thresholds, with a higher prevalence of hearing abnormalities in the high-frequency band (50.4%) than that of hearing abnormalities in the low-frequency band (20.9%). When the cumulative OP exposure was >1.118 score-years and the cumulative noise exposure was >88.9dB(A)-year, high-frequency hearing thresholds showed significant increases in pesticide-using farmers (32). A prospective study showed that the latency of waves I, III, and V of brainstem evoked response audiometry (BERA) was increased in the case of OP poisoning, demonstrating that significant changes occurred in both peripheral and central auditory pathways (33). From a case of combined intoxication of an aerosol containing malathion and methoxychlor (Ortho TM Orchard Spray) emerged a bilateral deep or permanent sensorineural hearing loss (SNHL) (34). In a cross-sectional study of Brazilian tobacco growers, central auditory dysfunction was found in the group exposed to OP compounds such as acetylmethamidophos and glyphosate, as evidenced by decrements in temporal processing and binaural integration processes (35). In a descriptive cross-sectional study of pesticide-exposed farmers in southern Brazil, more than half of the 40 respondents presented with hearing problems (36). Among the 18 rural workers exposed to organophosphate, 16 of them were found to present with irritant peripheral body balance disorders and 7 of them suffered from SNHL in a cross-sectional study, suggesting that OPs caused alterations in vestibular structures through a slow and silent poisoning (37). A study of occupationally exposed populations to OPs and pyrethroid pesticides showed that 56% of the exposed workers suffered from hearing loss at the central level (OR = 7.85, 95% Cl 2.9–19.8) compared to the unexposed group (38). Taken together, exposure to these insecticides could induce damage to the auditory system.

**Table 1:** Epidemiological studies on the effects of exposure to organophosphorus pesticides (OPs) on human auditory system.

**OPs**	**No. of subject**	**Country**	**Type of study**	**Co-exposure to noise**	**Methods**	**Result**	**Reference**
Mixture	16246	Iowa or North Carolina in USA	Cohort study	Not specified	Self-report	Compared with no exposure,the odds ratio for the highest quartile of exposure was 1.17 (1.03 to 1.31) for OPs; and 1.11 (0.99–1.24) for fonofos.	(29)
Mixture (methyl parathion, chlorpyrifos, diethyl succinate)	127	The Pontal do Paranapanema region in Brazilian	The observational, prospective, and cross-sectional study	Not specified	Pure tone audiometry, Vocal audiometry, Immittance testing	Auditory complaint and tinnitus with high pitch, had a higher incidence (*p* < 0.05) in groups exposed to pesticides in comparison to the control group	(31)
Mixture (insecticides, herbicides, and fungicides)	175 conventional farmers and 176 organic farmers	Three agricultural areas in Thailand	Cross-sectional study	Agricultural noise exposure	Standard pure tone audiometry (PTA); An interview	The highest category of cumulative insecticide exposure (score-years), cumulative OPs exposure (score-years) and cumulative noise exposure (dB(A)-years) were associated with an increased high-frequency band hearing threshold among conventional farmers	(32)
Organophosphorus compound (OPC)	100 patients of OPC poisoning	India	Prospective observational study	Not specified	Distortion Product Oto Acoustic Emission (DPOAE); Brain evoked response audiometry (BERA)	DPOAE was absent in 17 patients with respiratory failure and 51 patients without respiratory failure. BERA showed significant prolongation in wave I, wave III and wave V latencies in both the groups.	(33)
Mixture (organophosphates)	18 rural workers	Teresópolis, RJ in Brazilian	Cohort cross-sectional study	Not specified	Otorhinolaryngological, audiological, and vestibular examination; Questionnaires.	Sixteen workers had irritative peripheral body balance disorder and seven workers had sensorineural hearing loss. Agricultural pesticides cause vestibular alterations through a slow and silent intoxication.	(37)
Organophosphates and pyrethroid insecticides	98 workers exposed to insecticides and 54 non-exposed workers	Brazil	Cohort study	Not specified	An interview; Frequency patterns and duration patterns testing	Relative risk for hearing dysfunction was 7.58 for the exposure group (95% CI 2.9–19.8) in comparison with the non-exposed group.	(38)
Mixture (DDT and BHC organophosphates)	98	Brazil	Cross-sectional prevalence studies	Noise	Tonal audiometric test	The intensity of hearing loss in the group exposed to insecticides and noise was higher than in the group exposed to insecticides. The mean values of hearing loss intensity were increasing from 2 to 6 kHz, and decreasing by 8 kHz in relation to 6 kHz.	(39)
Malathion	120	Not mentioned	Retrospective study	With noise	Audiological interview; threshold tonal audiometry; Immittance measures	The group with concurrent exposure to noise and pesticide presented a higher incidence of hearing loss than group exposed to noise alone.	(40)
Mixture (organophosphates and pyrethroids)	56	Brazil	Cross-sectional cohort study	With noise	Pure-tone audiometry; acoustic immittance measurement; Brainstem auditory evoked potentials (BAEP); Dichotic digits test (DDT) assessment; Transient evoked otoacoustic emissions (TEOAE)	Compared with control group, the absolute latency of wave III and wave V was delayed, while the latency between peaks I—III and I—V increased at 2–4 kHz in the group exposed to Insecticides.	(41)
Mixture (chlorpyrifos, diazionon, fonofos, malathion, parathion-ethyl, parathion-methyl, profenofos, terbufos)	116 infants	China	Cohort study	Not specified	ABR	Exposure to multiple pesticides prenatally caused longer central conduction time and wave V latencies	(44)
Organophosphate insecticide	359 healthy women and 237 of their infants	China	Cohort study	Not specified	ABR	Many infants missed ABR wave I and III data at 18 months (*n* = 132); Methamidophos exposure was significantly different among infants with and without 18-month ABR data	(45)

Excessive noise exposure is one of the most common causes of hearing loss. It has been verified that the synergistic combination effect of OPs and noise exposure could result in more severe damage to the auditory system (39, 40). Guida et al. found that the percentage of people with hearing loss exposed to single-factor noise was 42.5%, while the percentage of people exposed to both malathion and noise exceeded 60% (40). A cross-sectional cohort study was carried out to assess the central auditory functions of endemic disease control agents. The results showed that there were significant intergroup differences in wave III and V absolute latencies, and interpeak I–III and I–V latencies bilaterally, with worse results appearing in the co-exposure group (occupational noise and pesticide) (41). These results indicate that OP exposure may bring about some brainstem neurotoxic effects and affect neuronal synapses located between the cochlear nucleus and the lateral lemniscus terminals (42, 43).

Exposure to OPs during embryonic life could cause neurodevelopmental toxicity, resulting in persistent brain neurotransmitter metabolism disorders, cognitive–behavioral abnormalities, and structural–morphological damage to brain tissue, as well as a negative impact on the auditory system. In a cohort study of Chinese infants, the ABR analysis of hearing in 9-month-old infants found that those prenatally exposed to multiple pesticides had a longer central conduction time, wave V latency, and negative effects on myelin-related gene expression, function, and myelination in the brain and the auditory system early in life (44). A study about early-life OPs exposure showed that five OPs were detectable in 10% of infant cord blood samples, including dibromophos, methamidophos, trichlorfon, CPF, and methomyl; maternal exposure to methamidophos during pregnancy resulted in a significant difference in ABR waves I and III in 18-month-old infants and an increased risk of hearing impairment (45).

To sum up, hearing damage can occur in the OP-exposed population, especially in the occupational population, presenting as hearing difficulties, persistent bilateral tinnitus, higher prevalence of hearing abnormalities, significantly increased incidence of hearing abnormalities, significant changes or dysfunction of the peripheral and central auditory system, increased high-frequency hearing thresholds, permanent SNHL, and alterations in vestibular structures. The combined exposure to OPs and occupational noise can exacerbate the degree of hearing loss in workers. Low-level pesticide exposures (partially OPs) in pregnant women and young children showed neurodevelopmental toxicity and abnormalities in ABR. These results mean that OP exposure is associated with adverse health effects for the auditory system in population studies.

## Experimental animal research

Animals used for hearing loss research mainly include rats, mice, guinea pigs, pigs, and squirrel monkeys (Table 2). The cochlear turns, the sensory hair cells (HCs), and the central auditory system of rodents are similar to those of humans in terms of auditory anatomy (46). Acquired hearing loss is related to the dysfunction of sensory elements in the inner ear, such as the impairment of sensory HCs and degeneration of spiral ganglion neurons (SGN) (47). Sensory HCs in the cochlea are mechanoreceptors that transduce the incoming sound information into electrical signals that are sent to the brain, while SGN is believed to innervate cochlear HCs (48). OP ototoxicity can be evaluated by an audiological test and histomorphological analysis of auditory brainstem implant on an experimental animal level. Finkler et al. found that the intraperitoneal (ip) injection of methamidophos in guinea pigs caused morphological alterations in their cochlea and injuries in the three turns, displaying as cilia distortion, derangement of the W pattern, and shortening of the cilia or yet, its absence. The longer the exposure to methamidophos, the greater the damage caused to the HCs. The damage site was mainly located in the outer HCs, developing from the parietal gyrus to the basal gyrus of the cochlea, with the first row of outer HCs being damaged at the turn, followed by the second and third rows of HCs (49). Reischl et al. found that squirrel monkeys showed a significant (*p* < 0.025) increase in daily hearing tests at 6 levels between 500 and 16,000 Hz after being exposed to 0.1 mg/kg of parathion for 148 days (50). The effects of methamidophos exposure on the vestibulocochlear system revealed cochlear morphological alterations, as well as cilia alterations in the saccule and the utricle, with dose intensification efficacy. Thus, methamidophos caused acute damage to the vestibulocochlear system (51). Bergler et al. found that a threshold shift of 10–20 dB and a prolongation of the interpeak latencies Jewett I–V of approximately 1 ms occurred after the application of paraoxon with a high dose of 27 mg/kg body weight in mini pigs (52). Upon subchronic inhalation of dichlorvos in Wistar rats, the exposed and control groups had similar plasma cholinesterase (ChE) activities on the level of ChE non-inhibition; however, distortion product otoacoustic emission (DPOAE) measurements showed a significant decrease at 8 kHz and 10 kHz in the exposed group; photomicrography observation showed that the damage caused by dichlorvos to the cochlea was similar to that induced by cisplatin, a known ototoxic drug (53). Histopathological analysis of the vestibular system of guinea pigs continuously exposed to CPF for 10 days showed no difference in the number of ciliary tufts of the saccule and the utricle maculae, whereas the trend with a borderline significance (*p* = 0.0569) was observed for the variable saccule. These results emphasize that OPs have neuro-ototoxic potential, but the dose and duration of exposure that are considered safe cannot be determined at this time (54). Soman, an organophosphorus nerve agent, is an irreversible ChE inhibitor. A study of soman-induced cerebral damage in rats showed that a significant decrease in DPOAE intensities was observed compared to controls (multivariate analysis of variance (MANOVA); F_(2.35)_ = 7.87; *p* = 0.002), and the amplitude of the DPOAE drop was well related to the severity of neuropathological consequences. Cochlear histology analysis showed that the alteration was present in spiral ganglion rather than the hair cells of Corti in the soman-exposed group. The nucleus of the damaged spiral ganglion was sometimes invisible or occurred strongly retracted and surrounded by large vacuoles (55, 56). Overall, OP exposure may decrease the hearing threshold or cause damage to cochlear HCs or SGN in experimental animals, leading to SNHL. By establishing the animal model of hearing loss, the ototoxic mechanism of OPs can be studied in depth.

**Table 2:** Experimental animal studies on the effects of exposure to organophosphorus persticides (OPs) on animal auditory system.

**OPs**	**Dose**	**Duration**	**Animals**	**Numbers of animals**	**Analytic methods**	**Result**	**Reference**
Methamidophos	0.3, 3.0 mg/kg/day	7 days	Guinea pigs	5 for control group; 16 for exposed group	Surface and electron microscopy(SEM)	In exposed group, the OHC cilia was absent, the W pattern of the cells was deranged and cilia was folded.	(49)
Parathion	0.1 mg/kg/day	148 days	Squirrel monkeys	4 for control group; 4 for exposed group	Hearing threshold	The parathion-exposed group showed a significant (*p* < 0.025) increasing at 6 levels between 500 and 16,000 Hz after a treatment of 40 days	(50)
Metamidophos	0.3, 3.0 mg/kg/day	7 days	Guinea pigs	3 for control group; 12 for exposed group	SEM	In exposed group, OHC cilia were absent or disarrayed in a “V” pattern, or folded, or one of the “V” arms was partially absent	(51)
Paraoxon	27 mg kg/BW	90 min	Mini pigs	1 for control group; 11 for exposed group	ABR	A prolongation of the interpeak latencies Jewett I-V of approximately 1ms; A threshold shift of 10–20 dB.	(52)
Dichlorvos	cisplatin (10 mL /kg); dichlorvos (8 mg/kg)	3 days	Wistar rats	8 for control group; 8 for positive control group; 8 for exposed group	DPOAE	Losses at frequencies of 8 and 10 kHz bilaterally were significant in the insecticide group. A significant difference of the control in 10 kHz on the right, as well as 8 and 10 kHz on the left ear exist.	(53)
Chlorpyrifos	0.5,1.0 mg/kg/day	10 days	guinea pigs	5 for control group; 3 for exposed group	SEM	The altered bulbous sacs were considered critical and showed a tendency to be significantly different.	(54)

## Exploration of mechanisms of hearing loss caused by OPs

**Figure 1:**
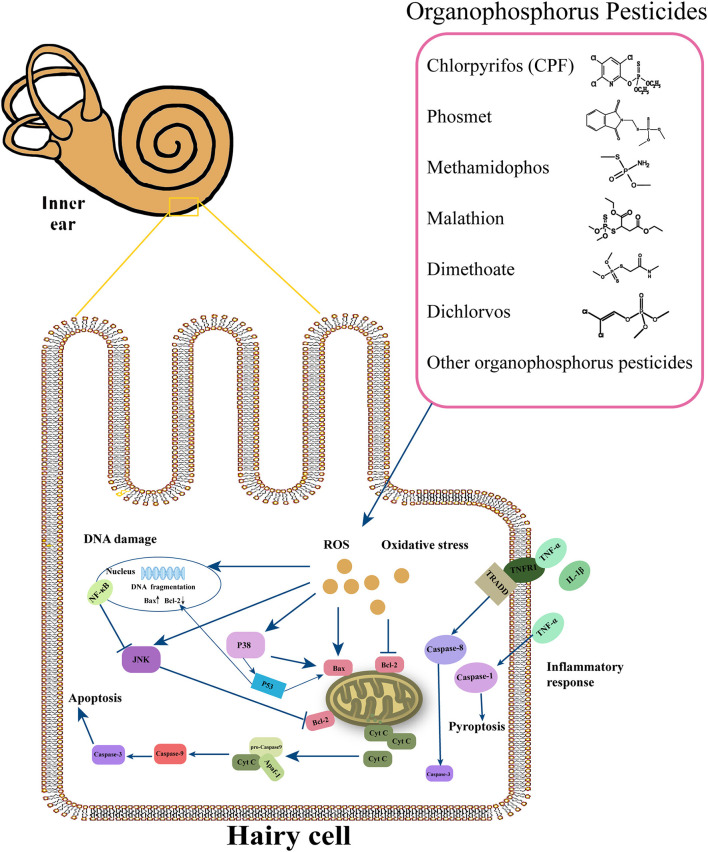
Overview of the major molecular events involved in cochlear damage produced by organophosphorus pesticides (OPs). This figure summarizes the possible molecular mechanisms under the OPs, including oxidative stress, DNA damage, and inflammatory response.

It has been demonstrated that the molecular mechanisms in the pathogenesis of SNHL implicate oxidative stress, DNA damage, inflammation, ion homeostasis disruption, mitochondrial dysfunction, apoptosis, etc. Many factors, such as noise, ototoxic drugs, environmental toxins, age, etc., promote edema of the stria vascularis and secrete cytokines and chemokines in the spiral ligament, leading to cochlear hypoxia and inflammatory response. These effects in turn may reduce the cochlear blood flow. Reactive oxygen species (ROS) and pro-inflammatory cytokines can move into hair cells (HCs), thus activating extrinsic and intrinsic apoptotic cascade responses. These responses cause mitochondrial dysfunction, which promotes the activation of the apoptotic cascade (57). Although population and experimental studies showed that OP-induced hearing loss mainly involves inner ear damage, the pathogenesis of hearing loss remains unclear. Next, we explored the mechanisms of cell death caused by OPs from oxidative stress, DNA damage, and inflammatory response (Figure 1).

### Oxidative stress induced by OPs

The term “oxidative stress” means cellular or tissue abnormalities owing to excess reactive oxygen species (ROS) production and exhausted antioxidant defenses (58). If the level of ROS production is beyond the resistant maximum threshold, organelle damage and even cell death will happen. Cochlear tissue is a highly active metabolic organ and can easily produce a large number of oxygen-free radicals in response to stressful conditions. Excess ROS production causes the peroxidation of cochlear lipids, thereby leading to cochlear cell death. Meanwhile, the generated vasoactive lipid peroxides will in turn reduce the blood flow of the cochlea, further leading to the reperfusion of the cochlear blood flow behind and generating a large amount of ROS. Oxidative damage can disturb the hair cell function into stria vascularis and spiral ligament fibrocytes (SLFs). ROS-producing enzymes (NOX2 and NOX3) have increased levels of noise/drug-induced hearing loss. OP exposure at minimal levels can generate large amounts of oxygen free radicals to attack intracellular macromolecules in animal experiments and on the cellular level (59). Also, OPs can increase mitochondrial calcium levels and excess accumulation of intracellular NO, leading to oxidative stress (60). Mitochondrial damage caused by OPs can also increase the production of ROS; excess ROS attacks biological membranes and induces lipid peroxidation, which, in turn, gives rise to mitochondrial dysfunction. Cytochrome C gets released from the mitochondria to the cytoplasm, which activates downstream effector caspase-3, resulting in programmed cell death (61). In goldfish (*Carassius auratus*), short-term exposure to the highly toxic monocrotophos resulted in the suppression of glutathione peroxidase (GPx) activity, increasing malondialdehyde (MDA) content, and generation of large amounts of ROS in a short duration, which in turn caused oxidative damage to DNA bases and telomeric DNA (62). In the cerebellum and cerebrum tissue of the F1 generation of Wistar rats following lactational exposure to CPF, lipid peroxidation exhibited a significant increase; the glutathione (GSH), respectively, declined by 49 and 51% in the cerebellum and cerebrum after maternal intoxication with CPF, which causes oxidative stress (63). In mouse-derived spermatogonial cell lines, Sertoli cell lines, and Leydig cell lines, the CPF treatment increased ROS and lipid peroxide production, decreased mitochondrial membrane potential, and increased levels of phosphorylated-adenosine monophosphate (AMP)-activated protein kinase, which is involved in cell apoptosis (64). Moreover, the environmental degradation product of CPF, TCP was readily enriched in aquatic fish bodies and can induce oxidative stress in zebrafish *Danio rerio* (65). In SH-SY5Y cells, the combined effect of CPF and cylindrospermopsin brought about a reduction in cell viability, an increase of intracellular GSH content, the enlargement of mitochondrial volume, and signs of apoptosis (66). CPF could provoke neuronal cell apoptosis by inducing ROS generation, which in turn activates the MAPK/JNK (mitogen-activated protein kinase/c-Jun NH2-terminal kinase) and p38 pathways (67). Subchronic exposure to dichlorvos in rats decreased superoxide dismutase (SOD) activity in mitochondria, caused lipid peroxidation, and caused cytochrome C (Cyt C) release from mitochondria into the cytoplasm, thus initiating the cells to enter apoptosis and ultimately leading to cell death (68). The effect of oxidative stress induced by dichlorvos could be inhibited by the mitochondria-targeted antioxidant MitoQ, thereby protecting cells from death (61). Exposure to trichlorfon in silver catfish (*Rhamdia quelen*) ruptured the blood–brain barrier (BBB), elevated brain ROS and brain lipid peroxides, reduced brain cell viability, and caused brain oxidative damage (69). Five OP (acetyl formate, methamidophos, chloramphos, malathion, and maloxon) treatment on PC12 cells showed that intracellular levels of ROS and malondialdehyde (MDA) were increased, while the activities of antioxidant enzymes, such as SOD, catalase, and glutathione peroxidase (GPx), were reduced. Furthermore, these effects of damage could be mitigated to a large extent by vitamin E pretreatment, suggesting that the cytotoxicities of OPs were mediated through oxidative stress (70). It is evident that OPs cause oxidative stress, which is a critical event of cell damage.

### DNA damage induced by OPs

Genomic DNA is constantly under assault by various environmental and intracellular factors that damage its chemical structure. Once the DNA is irrevocably damaged, cellular propagation may be impeded, and even cell death will happen (71). Previous studies have verified that ROS may directly react with DNA to form the 8-hydroxy-2′-deoxyguanosine (8-OHdG) DNA adduct. The caspase-mediated cell death pathways could lead to DNA damage *via* activation of the DNA fragmentation factor enzyme. Apoptosis-inducing factors could translocate to the nucleus to damage the genomic DNA (72). DNA damage is considered to be one of the possible mechanisms of hearing loss induced by noise, cisplatin, or other ototoxic compounds to cochlea cells.

OPs, such as acephate, methamidophos, malathion, and malaoxon, could directly or indirectly act on chromosomal DNA, leading to breakage in the single strand, cleavage of chromatin DNA into internucleosomal fragments, thus presenting the genotoxic potential (70). The combined effects of various OPs resulted in more severe DNA damage. CPF could induce DNA damage in HeLa and HEK293 cells, and the extent of single-stranded DNA breaks showed a significantly positive dose—response relationship and time dependence. The same roles were confirmed in Drosophila S2 and HepG2 cells (73, 74). CPF could result in DNA damage in rats by activating anti-inflammatory, antioxidant, and pro-apoptotic responses (75). However, CPF showed significant genotoxicity after 4h treatment of human lymphocytes without significant concentration dependence. The DNA damaging effects may be caused by a direct interaction of DNA with CPF and/or its metabolite that destabilized the DNA structure, rather than through free radicals due to no significant alteration in oxidative stress biomarkers (76). Exposure to dimethoate in rat bone-marrow cells led to significant micronuclei induction and metaphase chromosome abnormalities. Also, dimethoate significantly decreased the mitotic index and mitochondrial membrane potential, inhibited cyclin A2, and caused DNA damage, ultimately led to the apoptosis of leukocytes. These results confirmed that dimethoate showed immunotoxicity, genotoxic and cytotoxic potential (77). In the root cells of *Allium cepa* and *Vicia faba*, DNA damage and programmed cell death were observed after treatment with fenthion and malathion (78). Edifenphos, a broad-spectrum OP fungicide, has been demonstrated to accumulate in agricultural products and poses a hazard to human health. Edifenphos inhibited cell viability and induced DNA damage in lymphocytes. In addition, edifenphos caused ROS generation, which acts as an upstream signal, resulting in DNA damage and apoptosis in lymphocytes (79). In addition, the activation of the homologous recombination and non-homologous end-joining pathway is responsible for DNA damage. Diazinon could significantly enhance the messenger RNA (mRNA) level of homologous recombination pathway, including ataxia telangiectasia mutated (ATM), ataxia telangiectasia and Rad3-related protein (ATR), MRE11A, RAD51, breast cancer 1, and CHEK1 (Checkpoint kinase 1), whereas diazinon significantly reduced the mRNA level of non-homologous end-joining pathway. Moreover, diazinon also increased TP53 protein, which was a pro-apoptotic component of the DNA damage response. Summarily, diazinon exposure could cause DNA damage in granulosa cells (80). Fenitrothion, a broad-spectrum OP insecticide, raised the level of oxidative DNA damage by the elevation of 8-OHdG concentration in cerebrum and spleen tissues of male rats (81). Taken together, OPs can cause DNA fragmentation through cascade reactions following oxidative stress, or damage DNA by directly chemical-forming compounds with DNA, leading to cell death.

### Inflammatory response induced by OPs

Following various other traumas to the inner ear, the stria vascularis and spiral ligament express and release the tumor necrosis factor (TNF-) α, which promotes a robust inflammatory response and activation of the downstream pro-apoptotic signaling pathway. High levels of interleukin 1 beta (IL-1β) have also been detected in cochlea, promoting the formation of a pyroptosome or inflammasome that mediates caspase-1-dependent cell death *via* pyroptosis (82).

The phosmet and CPF exposure resulted in an increase of the inflammatory factor TNF-α in trophoblast JEG-3 cells and also induced the expression of interleukin 6 (IL-6) (83). CPF activated an immune system in the cortical tissue of rats, IL-1β and TNF-α, pro-inflammatory cytokines, and released increasingly in the brain from astrocytes, microglia, and Schwann cells (84). CPF enhanced the secretion of the pro-inflammatory mediators by activating the nuclear factor kappa B (NF-κB) in the brain tissue of neonatal rats (85). Dietary exposure to CPF in colitic mice resulted in higher percentages of circulating neutrophils and mast cells. A shortened length, tissue edema, and lipid peroxidation of the colon were also observed. It was speculated that CPF affected immune cell populations and activated inflammatory responses, leading to a more severe tissue damage in mice with dextran sulfate-induced colitis (86, 87). The expression levels of TNF-α, interleukin 10 (IL-10), and transforming growth factor (TGF)-β were upregulated in the liver; the numbers of blood neutrophils and hepatic neutrophils were increased in a low-dose CPF-exposed diabetic mouse model. Dietary exposure to CPF affected the distribution of myeloid and lymphoid immune cells in the blood and liver of diabetic mice, which may lead to excessive inflammation (88). BV-2 microglial cells treated with CPF overexpressed inflammatory cytokines (IL-1β and NLRP3 [NOD-, LRR-, and pyrin domain-containing protein 3)] (89). Exposure to CPF in neonatal rats elevated the expression levels of pro-inflammatory factors (such as IL-6 and TNF-α), as well as the activation of HMGB1 (high mobility group box 1 protein), a DNA-associated non-histone protein that is considered to be a late-stage inflammatory factor in many neurodegenerative diseases (85). Subchronic exposure to dimethoate elevated mRNA levels of TNF-α and IL-6, and enhanced the striatal neuroinflammatory response in the hippocampus and the striatum of male mice (90). After intoxication with dimethoate for 5 weeks, the mRNA levels of TNF-α and IL-6 increased significantly in the hippocampus, while the proportion of Iba1 immunoreactive cells was also increased. Under a low dose of dimethoate—comparable to the levels of the pesticide present as residues in food, the subchronic exposure brought about a pro-inflammatory status in the brain and triggered a neuroinflammatory response to the lipopolysaccharide challenge with regional specificity (91). Inflammatory responses are regulated by both pro-inflammatory and anti-inflammatory cytokines that are related to the exposure time of OPs. In adult male mice, after exposure to diisopropylfluorophosphate (DFP) for 1 h, there was an increase of pro-inflammatory markers (Il1b, Tnf), but lack of immunoregulatory (Il1rn, Il4ra, Socs3) and anti-inflammatory marker (Lgals3, Mrc1, Igf1, Il10) modulations. Microglial cells presented a pro-inflammatory phenotype very rapidly, while the immunoregulatory marker started upregulation after DFP exposure for 1 h (92). Besides, the intake of dichlorvos at low doses caused weight loss and increased tumor mass in solid Ehrlich tumor-bearing mice, which were associated with high levels of hydroge peroxide (H_2_O_2_) production and TNF (93).

The cohort research of two distinct populations from Pakistan and Cameroon found that the levels of IL-6, IL1-β, and TNF-α in chronically OP-exposed groups were higher than those in the non-exposed group, with a statistically significant increase. The paraoxyglucose Paraoxonase 1 (PON1), a serum esterase/lactonase, played a key role in the hydrolysation of various OPs. The catalytic ability of PON1 was mainly affected by rs662 and rs854560 single-nucleotide polymorphisms (SNPs). It was found that, in exposed individuals of both populations, rs662 SNP risk genotype had a causative association with acetylcholinesterase (AChE), PON1 activity levels, and pro-inflammatory biomarkers [IL-6, TNF-α, and C-reactive protein (CRP)] (94). In all, OPs could induce an inflammatory response to promote cell death.

## Discussion and outlook

OP insecticides have great advantages in pest control due to their low cost, high performance, and broad spectrum. However, they also increase insecticide resistance, pose a risk to human health, pollute the environment, and so on. About 3 million poisoning cases and approximately 250,000 deaths occur annually worldwide because of pesticides, where most of the deaths are caused by OPs (95). Numerous studies demonstrated that chronic OP exposure is associated with high genotoxicity, embryotoxicity, neurotoxicity, metabolism disorders, etc. However, little attention has been paid to the effects of OP exposure and hearing loss. Hearing loss is one of the major health concerns and seriously affects the life quality of humans all over the world. There are many causative factors for hearing loss, of which SNHL is responsible for the majority (96). Besides known factors, such as age, noise, ototoxic drugs, metals, and genetic factors, potential risk factors from the environment should be worthwhile to mine. This is the first systematical review to analyze the association between OP exposure and hearing loss from both population-based studies and animal experiments; furthermore, we attempted to probe into the mechanism responsible for cell damage caused by OPs.

By reviewing the papers about epidemiological population studies, we concluded that hearing damage could occur in the OP-exposed population, especially in the occupational population. With ABR and OAE testing on humans, both peripheral and central auditory systems appear to be dysfunctional. However, population-based studies are limited in several ways. OPs are the largest and most diverse types of pesticides, so it is difficult to locate which OPs would be responsible for hearing loss in population studies. Moreover, the sources of OP exposure are varied and complex. It is difficult to assess the dose arising from environmental and occupational exposure (97). OPs are non-persistent with short half-lives, the damaging effects of which could be detected only after a longlasting exposure. Many confounders that contribute to hearing loss make it relatively difficult to judge the ototoxic roles of OPs based on population studies. Additionally, the effect of a certain OP can be evaluated based on animal models. In experimental animal studies, only several kinds of OPs (methamidophos, paraoxon, dichlorvos, chlorpyrifos, and parathion) were determined to exhibit some degree of ototoxicity. Similar to the ototoxicity of aminoglycoside antibiotics and cisplatin, OP exposure resulted in morphological alterations in cochlea, threshold elevation, and prolongation of the interpeak latencies. However, the effects of OPs-induced hearing loss on experimental animals may be affected by animal species, dose, duration, and delivery route of agents (98). The exposure route in animal experiments could be different in humans, leading to an inconsistent outcome. In all, these results show that OP exposure is associated with hearing loss. Due to the fact that there is no regeneration of destroyed sensory cells in the adult mammalian cochlea, hearing loss becomes an irreversible process. Therefore, the preventive strategy for OPs poisoning could be even more important.

In previous studies, auditory hairy cells may undergo cell death by apoptosis and necrosis under various insults. Several drugs, such as aminoglycosides and cisplatin, can initiate apoptotic cell death and DNA damage through oxidative stress, cytochrome c (Cyt c) release, and caspase-3 activation. Involved signaling pathways may include the upregulated expression of inflammatory cytokines and chemokines, as well as the generation of oxidative stress (82). These effects of several OPs were also confirmed on many cell lines or various models, such as goldfish, and root tip cells of plants. Oxidative stress, DNA damage, and inflammatory response may be mainly responsible for the cell death of auditory cells caused by OPs, which ultimately induce hearing loss. However, this hypothesis still needs further verification. Cell-based assay is a good system for the study of ototoxicity *in vitro*. The House Ear Institute-Organ of Corti 1 (HEI-OC1) represents a common progenitor for auditory cells and expresses several markers such as prestin, connexin 26, and myosin7a. The HEI-OC1 cell line is proposed as a useful model to investigate the biological responses associated with auditory cells (99). Currently, HEI-OC1 cells were successfully used to evaluate the ototoxicity of several HIV (human immunodeficiency virus) retroviral agents. The results showed that several drugs, such as efavirenz, ritonavir, delavirdine, nelfinavir, and tenofovir, significantly reduced the viability of auditory cells (100). Also, it has been used to screen protective pharmaceuticals against ototoxicity caused by aminoglycosides and cisplatin. Additionally, the mouse cochlear explant at postnatal days 3–5 was used to explore the ototoxicity of insecticide carbaryl and herbicide paraquat (101). In addition, Zebrafish (*Danio rerio*) is a useful auditory research model owing to its detailed perception of sound and easy accession of its lateral line. The zebrafish lateral line is an easy-to-screen putative ototoxic *in vivo* for understanding ototoxic mechanisms and developing protective agents. However, unlike auditory cell lines, each neuromast of zebrafish lateral line contains HCs and supporting cells, and each HC receives both afferent and efferent innervation (102).

## Conclusion

In summary, there is an association between hearing loss and OP exposure. This review recapitulates the main mechanisms of hearing loss caused by OPs including oxidative stress, DNA damage, and inflammation. It can provide a novel consideration for future studies of environmental pollutants in SNHL. OP exposure is a long-term and underlying process, so their effects may be imperceptible in a timely manner. Therefore, appropriate hearing protection measures should be brought forward, including short-interval audiometric examinations, efficient hearing protectors, and intervention by anti-inflammatory and antioxidant drugs.

## Author contributions

MSZ and MH: data collection and original draft. JYZ and RC: funding acquisition and writing-review and editing. RC: conceptualization and supervision. All authors contributed to the article and approved the submitted version.

## Funding

This work was supported by the Zhejiang Provincial Natural Science Foundation of China under Grant number LQ20B070006 and the National Natural Science Foundation of China (Grant Number 22106034).

## Conflict of interest

The authors declare that the research was conducted in the absence of any commercial or financial relationships that could be construed as a potential conflict of interest.

## Publisher's note

All claims expressed in this article are solely those of the authors and do not necessarily represent those of their affiliated organizations, or those of the publisher, the editors and the reviewers. Any product that may be evaluated in this article, or claim that may be made by its manufacturer, is not guaranteed or endorsed by the publisher.
